# Validation of Endometriosis Fertility Index for the Prediction of Non-Assisted Reproductive Technology (ART) Pregnancy in Women With Endometriosis-Associated Infertility: A Prospective Cohort Study

**DOI:** 10.7759/cureus.111159

**Published:** 2026-06-19

**Authors:** Divya Mishra, Shantanu Shubham, Neena Malhotra

**Affiliations:** 1 Obstetrics and Gynecology, Graphic Era (Deemed to be University), Dehradun, IND; 2 Neonatology, Graphic Era (Deemed to be University), Dehradun, IND; 3 Obstetrics and Gynecology, All India Institute of Medical Sciences, New Delhi, New Delhi, IND

**Keywords:** endometriosis, endometriosis fertility index, infertility, laparoscopy, pregnancy prediction

## Abstract

Introduction

Endometriosis is a common cause of infertility and affects reproductive outcomes through multiple mechanisms, including pelvic adhesions, altered pelvic anatomy, and inflammatory changes. The Endometriosis Fertility Index (EFI) was developed as a prognostic tool to predict spontaneous conception after the surgical treatment of endometriosis. This study aimed to validate the EFI for predicting non-assisted reproductive technology (ART) pregnancy outcomes after laparoscopic surgery in women with endometriosis-associated infertility and compare its predictive performance with the revised American Society for Reproductive Medicine (r-ASRM) classification.

Methods

A prospective cohort study was conducted in the Department of Obstetrics and Gynecology at All India Institute of Medical Sciences (AIIMS), New Delhi, between January 2016 and December 2017 with a six-month postoperative follow-up. Seventy-two women with endometriosis-associated infertility confirmed during operative laparoscopy were included. EFI scores were calculated postoperatively using historical parameters (age, the duration of infertility, and prior pregnancy) and surgical parameters (Least Function Score {LFS} and ASRM score). Patients underwent individualized fertility management, including expectant management, ovulation induction, and intrauterine insemination (IUI) where indicated. Receiver operating characteristic (ROC) analysis, Kaplan-Meier survival analysis, and Cox proportional hazards regression were performed to assess predictive accuracy.

Results

The mean EFI score was 6.7 ± 1.6. Women who achieved clinical pregnancy had significantly higher EFI scores than those who did not conceive (8.2 ± 1.2 versus 6.0 ± 1.3; p < 0.001). An EFI cut-off of ≥7 predicted clinical pregnancy with a sensitivity of 95.8% and specificity of 52.3%, with an area under the curve (AUC) of 0.90 (95% confidence interval {CI}: 0.82-0.97). The hazard ratio for conception among women with an EFI of ≥7 was 19.2 (95% CI: 2.6-142.1; p = 0.004). Women with EFI scores of 9-10 demonstrated a 100% pregnancy rate within six months. The cumulative probability of non-ART pregnancy at six months was 33.3%. An ASRM score of <21 also demonstrated predictive capability with an AUC of 0.92.

Conclusion

EFI demonstrated strong predictive performance for non-ART pregnancy outcomes following laparoscopic surgery in women with endometriosis-associated infertility. The index may serve as a valuable clinical tool for individualized fertility counselling, prognostic assessment, and guiding decisions regarding expectant management versus early assisted reproductive intervention.

## Introduction

Endometriosis is defined as the presence of endometrial-like tissue outside the uterine cavity and is associated with a chronic inflammatory response [1]. It affects approximately 6%-15% of women in the reproductive age group and is identified in 25%-50% of infertile women [2-5]. Infertility with endometriosis involves multiple factors, such as anatomical distortion, inflammation, altered folliculogenesis, impaired endometrial receptivity, and damaged tubal function [6,7]. Pelvic adhesions resulting from endometriotic lesions may disrupt the tubo-ovarian relationship and impair ovum pickup and transport [8]. Additionally, cytokines such as interleukins, tumor necrosis factor-alpha, and vascular endothelial growth factor alter the peritoneal environment, impacting oocyte quality and embryogenesis [6,7,9].

The clinical presentation of endometriosis is heterogeneous, ranging from minimal peritoneal deposits to severe pelvic adhesions and deep infiltrating disease [10-13]. Various classifications, such as the American Fertility Society in 1979 and the American Society for Reproductive Medicine (ASRM) in 1985, have been developed to standardize diagnosis and aid treatment planning, with the latter being the most widely used [13,14]. The ASRM scoring system categorizes disease severity based on laparoscopic findings, including peritoneal implants, ovarian endometriomas, and adhesions [13,15-17].

The Endometriosis Fertility Index (EFI) predicts pregnancy without assisted reproductive technology (ART) after the surgical treatment of endometriosis. The EFI incorporates historical factors, including age, the duration of infertility, and prior pregnancy history, as well as surgical factors, such as the Least Function Score (LFS) and ASRM score. The Least Function Score assesses the postoperative functional status of the fallopian tubes, fimbria, and ovaries, providing an estimate of reproductive potential following surgical correction [13]. The EFI correlates with pregnancy rates in studies and helps clinicians discuss fertility prognosis and need for assisted reproductive therapy [17-20]. Tomassetti et al. linked the EFI score to the time to non-ART pregnancy in women undergoing laparoscopic endometriosis surgery [21]. Wang et al. found EFI better than ASRM in predicting in vitro fertilization (IVF) pregnancy outcomes [3]. Zeng et al. showed higher cumulative pregnancy rates with increasing EFI scores, supporting its predictive power for natural conception [20].

More recent data support the EFI as a clinically useful prognostic tool in endometriosis-related infertility and suggest its use in individualized fertility counselling and treatment planning [22,23]. Contemporary research also highlights the potential integration of imaging findings and biomarkers with EFI to improve the predictive accuracy of fertility outcomes [24,25].

A 2020 systematic review in BJOG of 17 studies with 4598 women found that EFI has moderate-to-good predictive ability for spontaneous pregnancy after endometriosis surgery. It is the most validated tool for predicting pregnancy outcomes without assisted reproduction, with the Least Function Score being the key prognostic factor [26]. Due to heterogeneity among studies, prospective validation in populations other than those in which it was first developed is warranted.

The severity of endometriosis was assessed using the revised American Society for Reproductive Medicine (r-ASRM) classification system, which remains one of the most widely used surgical staging systems for endometriosis [15]. The Endometriosis Fertility Index (EFI), developed and validated by Adamson and Pasta, integrates historical factors, age, the duration of infertility, pregnancy history, and surgical factors, including the Least Function Score (LFS), to predict the likelihood of spontaneous conception following endometriosis surgery [13]. The Least Function Score represents the postoperative functional assessment of the fallopian tubes, fimbria, and ovaries and constitutes an important component of the EFI scoring system.

The present prospective cohort study was designed to validate the EFI in predicting non-ART pregnancy outcomes in women undergoing laparoscopic surgery for endometriosis-associated infertility in India. Additionally, the study aimed to compare the predictive ability of EFI with that of ASRM classification and to evaluate its role in estimating time to conception following surgical treatment.

This work was previously presented as a poster/abstract at Fertility 2019: Technologies and Controversies in Reproduction, 3-5 January 2019, International Convention Centre (ICC) Birmingham, United Kingdom.

## Materials and methods

Study design and setting

This prospective cohort study was conducted in the Department of Obstetrics and Gynecology at All India Institute of Medical Sciences (AIIMS), New Delhi, between January 2016 and December 2017, with a follow-up period of six months after laparoscopic surgery. All participants were prospectively followed for six months following laparoscopic surgery, and pregnancy outcomes occurring within this period were included in the analysis. The study aimed to assess the predictive performance of EFI on non-ART pregnancy outcomes in women with endometriosis-associated infertility. The Institute Ethics Committee for Post Graduate Research of All India Institute of Medical Sciences, New Delhi, issued approval IECPG-62/27.11.2015, RT-19/30.12.2015.

Study population

Women with infertility and suspected endometriosis were evaluated. Those undergoing operative laparoscopy with confirmed endometriosis were eligible. Diagnosis was made by laparoscopic visualization, with or without histopathology, the gold standard [4].

Inclusion and exclusion criteria

Women with surgically corrected endometriosis were included in the study if they were younger than 38 years of age, had a body mass index (BMI) between 18 and 28 kg/m², possessed at least one patent fallopian tube demonstrated on chromopertubation, had a normal uterine cavity, and had a male partner with normal semen parameters according to the WHO 2010 criteria [27]. Patients were excluded if they had a history of previous surgery for endometriosis, polycystic ovarian syndrome, genital tuberculosis confirmed by histopathology or culture, bilateral tubal blockage, male factor infertility, intrauterine adhesions, or a compromised uterine cavity.

Sample size calculation

Sample size was estimated based on previously published validation studies by Tomassetti et al. [21] and Wang et al. [3], which reported cumulative pregnancy rates ranging from 41% to 54%. Assuming an expected pregnancy rate of approximately 40%-50%, a minimum sample size of 60 participants was calculated to achieve 80% power with 5% level of significance for the study duration.

Surgical evaluation and scoring

All participants had laparoscopic surgery for endometriosis diagnosis and treatment. Disease severity was staged during the procedure using the revised ASRM classification based on peritoneal implants, ovarian endometriomas, and pelvic adhesions [13]. After surgical correction, the EFI score was calculated for each participant using both historical and surgical parameters as described by Adamson and Pasta [13]. Historical factors included age, infertility duration, and prior pregnancy history. Surgical factors encompassed ASRM total score, lesion score, and Least Function Score, which reflected postoperative reproductive potential of the fallopian tubes, fimbriae, and ovaries [13]. EFI scores ranged from 0 to 10, with higher scores indicating a better prognosis for spontaneous conception.

Follow-up protocol

Postoperatively, patients received expectant management or ovulation induction tailored to their profiles, including clomiphene citrate, gonadotropins, or both, based on age, ovarian reserve, and BMI. Follicular monitoring used transvaginal ultrasonography, and ovulation was triggered with human chorionic gonadotropin when the follicle reached ≥18 mm. Intrauterine insemination (IUI) was performed 36 hours after the ovulation trigger when indicated. All women were followed for a period of six months after surgery for the assessment of pregnancy outcomes for six months after surgery.

Outcome measures

Primary outcomes included clinical pregnancy, defined as the ultrasound visualization of an intrauterine gestational sac with fetal cardiac activity at ≥6 weeks of gestation; ongoing pregnancy, defined as the continuation of pregnancy beyond 20 weeks of gestation; and time to conception within six months following surgery.

Statistical analysis

Statistical analyses evaluated EFI and ASRM scores for pregnancy outcomes. Continuous variables were means ± SD, categorical and proportions. Receiver operating characteristic (ROC) curves assessed sensitivity, specificity, and predictive values for pregnancy, with the area under the curve (AUC) indicating performance. Kaplan-Meier estimated conception probability over time, tested via log-rank. Cox regression calculated hazard ratios across EFI categories. Odds ratios with 95% confidence intervals (CIs) assessed EFI cut-offs and outcomes. P < 0.05 was significant. Statistical analysis was performed using Stata software version 14.0 (StataCorp LLC, College Station, TX).

## Results

A total of 72 women with endometriosis-associated infertility were recruited from January 2016 at the AIIMS Gynecology Outpatient Department after undergoing laparoscopic surgery for suspected endometriosis. Intraoperative ASRM scores and postoperative EFI scores were calculated using historical and surgical parameters. All patients were followed for six months after surgery. The baseline characteristics of the study population are shown in Table 1.

**Table 1:** Demographic characteristics Data presented as mean ± SD (minimum-maximum) and n (%) BMI: body mass index

Variable (n = 72)	Summary measures
Age (years)	28.8 ± 3.9 (21-38)
≤35 years	69 (95.8)
>35 years	3 (4.2)
BMI (kg/m²)	23.9 ± 2.6 (18-28)
Duration of infertility (months)	38.6 ± 29.0 (12-144)
Years infertile	
≤3 years	51 (70.8)
>3 years	21 (29.2)
Infertility type	
Primary	57 (79.2)
Secondary	15 (20.8)

Most participants were younger than 35 years, with a mean age of 28.8 ± 3.9 years. The mean body mass index was 23.9 ± 2.6 kg/m². Among the 72 women included in the study, primary infertility was observed in 57 women (79.2%), and the mean duration of infertility was 38.6 ± 29.0 months. The mean ASRM endometriosis lesion score and total ASRM score were 21.7 ± 13.0 and 38.8 ± 29.0, respectively. Following surgical correction, Least Function Scores were categorized as high (7-8) in 19 (26.4%) patients, moderate (4-6) in 50 (69.4%) patients, and low (1-3) in three (4.2%) patients. Postoperative EFI scores ranged from 2 to 10, with a mean score of 6.7 ± 1.6. Patient characteristics related to ASRM and EFI scores are shown in Table 2.

**Table 2:** Patient characteristics related to ASRM and EFI scores Data presented as mean ± SD (minimum-maximum) and n (%) ASRM, American Society for Reproductive Medicine; EFI, Endometriosis Fertility Index

Variable (n = 72)	Summary measures
ASRM endometriosis score (0-58)	21.7 ± 13.0 (1-41)
ASRM endometriosis score	
≥16	57 (79.2)
<16	15 (20.8)
ASRM total score (0-178)	38.8 ± 29.0 (1-124)
ASRM total score	
≥71	14 (19.4)
<71	58 (80.6)
Least Function Score (0-8)	5.7 ± 1.5 (2-8)
Least Function Score	
High score (7-8)	19 (26.4)
Moderate score (4-6)	50 (69.4)
Low score (1-3)	3 (4.2)
EFI score (0-10)	6.7 ± 1.6 (2-10)

We compared the postoperative Endometriosis Fertility Index (EFI) scores between women who conceived within six months after surgery and those who did not. Women who achieved clinical pregnancy had significantly higher mean EFI scores than those who did not conceive (8.2 ± 1.2 versus 6.0 ± 1.3; p < 0.001), indicating a strong positive correlation between higher EFI scores and improved pregnancy outcomes.

Similarly, American Society for Reproductive Medicine (ASRM) scores were compared between the two groups. Women who conceived had significantly lower mean ASRM scores compared to those who did not achieve pregnancy (12.8 ± 14.4 versus 51.8 ± 25.6; p < 0.001). These findings suggest that lower ASRM scores were associated with a greater likelihood of conception within six months following surgery. The correlation of EFI and ASRM scores with clinical pregnancy outcomes is shown in Table 3.

**Table 3:** Correlation of EFI and ASRM scores with clinical pregnancy Data presented as mean ± SD and p < 0.05 is statistically significant ASRM, American Society for Reproductive Medicine; EFI, Endometriosis Fertility Index

Variable	Total score	With clinical pregnancy	Without clinical pregnancy	P value
EFI score	6.7 ± 1.6	8.2 ± 1.2	6.0 ± 1.3	<0.001
ASRM score	38.8 ± 29.0	12.8 ± 14.4	51.8 ± 25.6	<0.001

Receiver operating characteristic (ROC) analysis was performed using an Endometriosis Fertility Index (EFI) score cut-off of ≥7 and an American Society for Reproductive Medicine (ASRM) score cut-off of <21 for the prediction of clinical pregnancy. For an EFI of ≥7, the sensitivity and specificity for predicting clinical pregnancy were 95.8% (95% CI: 79.8-99.3) and 52.3% (95% CI: 37.9-66.3), respectively, with an area under the curve (AUC) of 0.90 (95% CI: 0.82-0.97).

For an ASRM score of <21, the sensitivity and specificity were 83.3% (95% CI: 64.2-93.3) and 79.2% (95% CI: 65.7-88.3), respectively, with an AUC of 0.92 (95% CI: 0.86-0.98). The ROC analysis for the prediction of clinical pregnancy using EFI and ASRM score cut-offs is shown in Table 4.

**Table 4:** Cut-offs of EFI and ASRM scores against clinical pregnancy using ROC analysis ASRM, American Society for Reproductive Medicine; AUC, area under the curve; CI, confidence interval; EFI, Endometriosis Fertility Index; FN, false negative; FP, false positive; NPV, negative predictive value; PPV, positive predictive value; TN, true negative; TP, true positive; ROC, receiver operating characteristic

Cut-off	EFI score (≥7)	ASRM score (<21)
TP	23	20
FP	21	10
TN	27	38
FN	1	4
Sensitivity (95% CI)	95.8% (79.8-99.3)	83.3% (64.2-93.3)
Specificity (95% CI)	52.3% (37.9-66.3)	79.2% (65.7-88.3)
PPV (95% CI)	52.3% (37.9-66.3)	66.7% (48.8-80.8)
NPV (95% CI)	95.8% (79.8-99.3)	90.5% (77.9-96.2)
AUC (95% CI)	0.90 (0.82-0.97)	0.92 (0.86-0.98)

**Figure 1:**
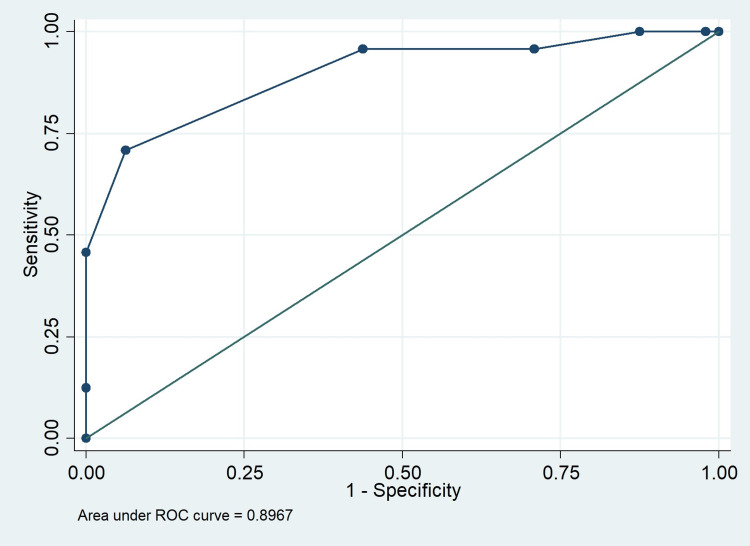
Correlation between EFI score and clinical pregnancy using ROC analysis The small blue circles represent the individual sensitivity and specificity coordinate points obtained at different cut-off values of the respective scoring systems on the ROC curve. The solid blue line connecting these points represents the ROC curve, illustrating the diagnostic performance of the score across varying thresholds. The diagonal straight line represents the line of no discrimination (reference line), indicating the performance expected by chance alone (area under the curve = 0.5) EFI, Endometriosis Fertility Index; ROC, receiver operating characteristic

**Figure 2:**
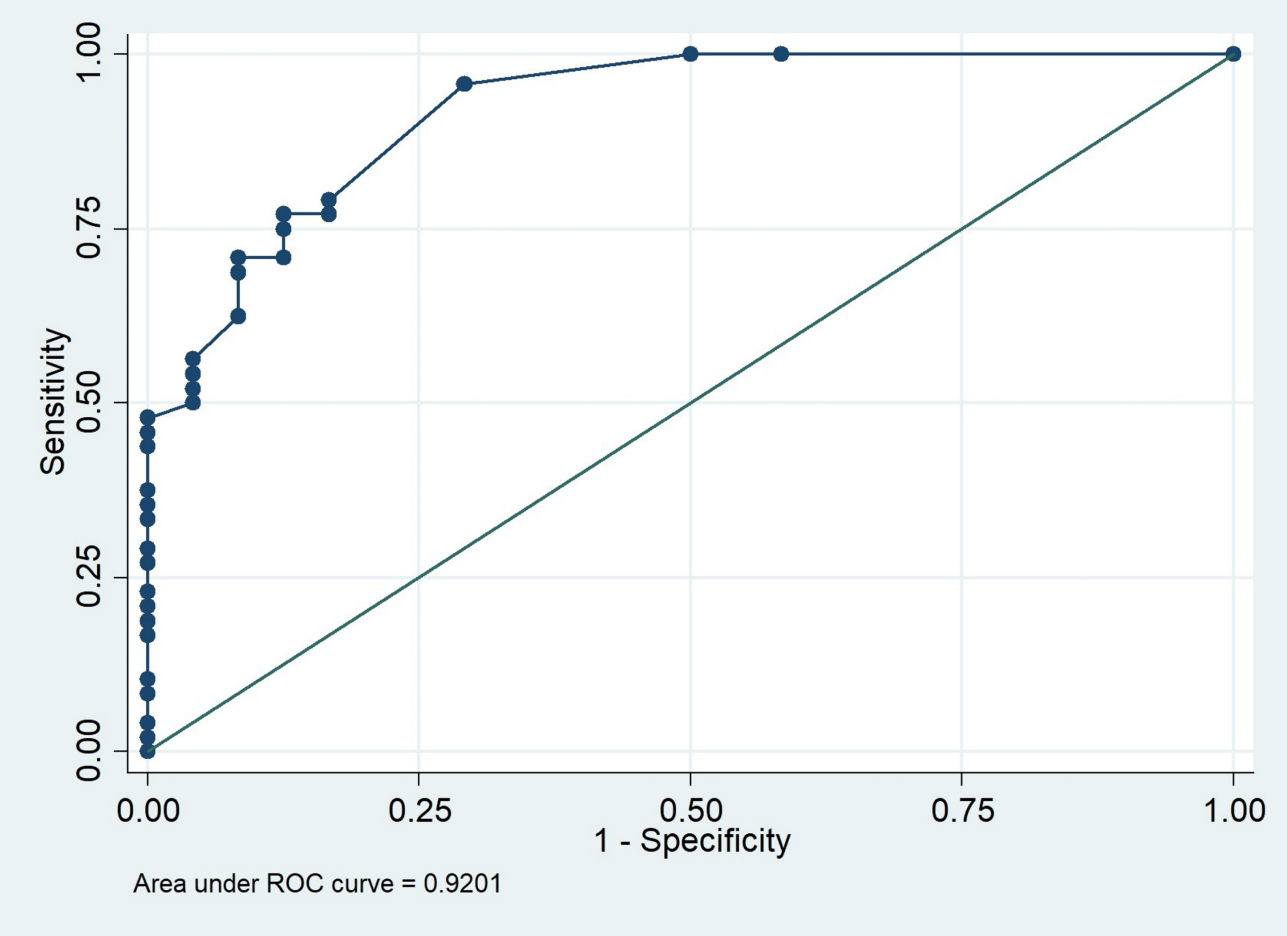
Correlation between ASRM score and clinical pregnancy using ROC analysis The small blue circles represent the individual sensitivity and specificity coordinate points obtained at different cut-off values of the respective scoring systems on the ROC curve. The solid blue line connecting these points represents the ROC curve, illustrating the diagnostic performance of the score across varying thresholds. The diagonal straight line represents the line of no discrimination (reference line), indicating the performance expected by chance alone (area under the curve = 0.5) ASRM, American Society for Reproductive Medicine; ROC, receiver operating characteristic

As shown in Table 4, the EFI score cut-off of ≥7 demonstrated higher sensitivity and negative predictive value compared to the ASRM score cut-off of <21. However, the area under the curve (AUC) values from receiver operating characteristic (ROC) analysis were comparable for both scores, suggesting similar predictive ability for clinical pregnancy in the study population. Both EFI and ASRM score cut-offs showed statistically significant associations with clinical pregnancy outcomes (p = 0.001 and p < 0.001, respectively). The ROC curves for EFI and ASRM scores in relation to clinical pregnancy are shown in Figure 1 and Figure 2, respectively.

The same EFI and ASRM score cut-offs were used for predicting ongoing pregnancy outcomes. For an EFI score cut-off of ≥7, the sensitivity and specificity were 94.7% (95% CI: 75.4-99.1) and 50.9% (95% CI: 37.9-63.9), respectively, with an AUC of 0.86 (95% CI: 0.76-0.96). Similarly, using an ASRM score cut-off of <21, the sensitivity and specificity for predicting ongoing pregnancy were 84.2% (95% CI: 62.3-94.5) and 73.6% (95% CI: 60.4-83.6), respectively, with an AUC of 0.87 (95% CI: 0.79-0.96). The predictive performance of EFI and ASRM score cut-offs for ongoing pregnancy is shown in Table 5.

**Table 5:** Cut-offs of EFI and ASRM scores against ongoing pregnancy using ROC analysis ASRM, American Society for Reproductive Medicine; AUC, area under the curve; CI, confidence interval; EFI, Endometriosis Fertility Index; FN, false negative; FP, false positive; NPV, negative predictive value; PPV, positive predictive value; TN, true negative; TP, true positive; ROC, receiver operating characteristic

Cut-off	EFI score (≥7)	ASRM score (<21)
TP	18	16
FP	26	14
TN	27	39
FN	1	3
Sensitivity (95% CI)	94.7% (75.4-99.1)	84.2% (62.3-94.5)
Specificity (95% CI)	50.9% (37.9-63.9)	73.6% (60.4-83.6)
PPV (95% CI)	40.9% (27.7-55.6)	53.3% (36.1-69.8)
NPV (95% CI)	96.4% (82.3-99.4)	92.9% (81.0-97.5)
AUC (95% CI)	0.86 (0.76-0.96)	0.87 (0.79-0.96)

**Figure 3:**
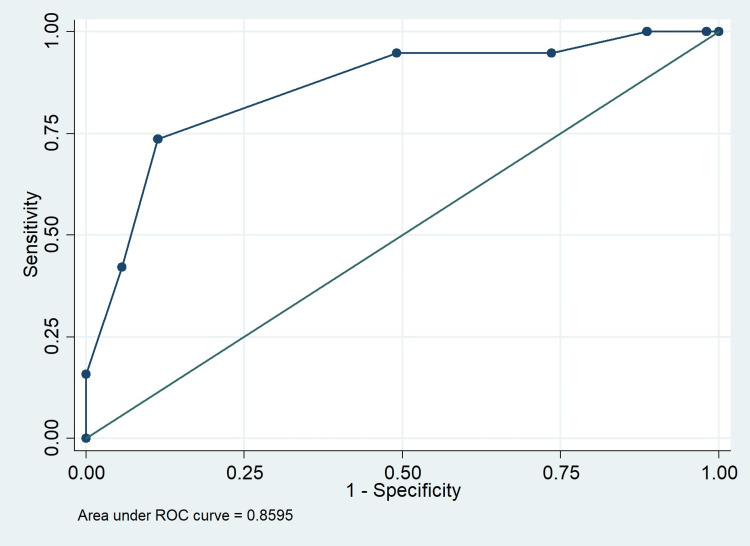
Correlation between EFI score and ongoing pregnancy using ROC analysis The small blue circles represent the individual sensitivity and specificity coordinate points obtained at different cut-off values of the respective scoring systems on the ROC curve. The solid blue line connecting these points represents the ROC curve, illustrating the diagnostic performance of the score across varying thresholds. The diagonal straight line represents the line of no discrimination (reference line), indicating the performance expected by chance alone (area under the curve = 0.5) EFI, Endometriosis Fertility Index; ROC, receiver operating characteristic

**Figure 4:**
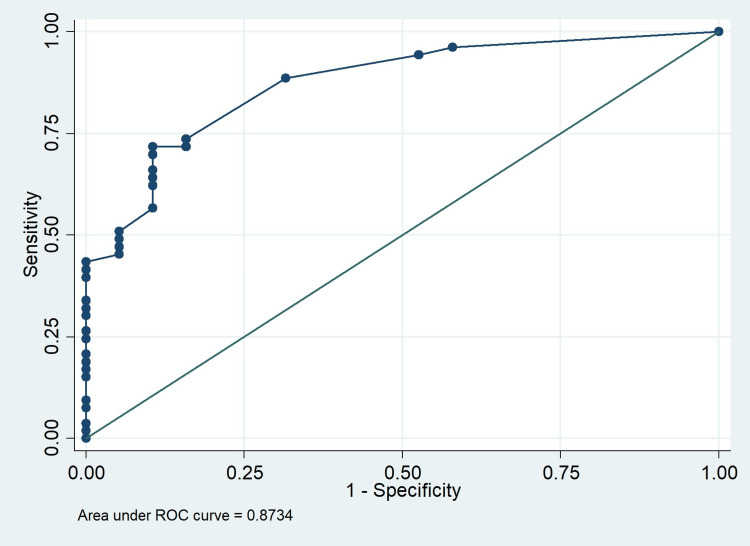
Correlation between ASRM score and ongoing pregnancy using ROC analysis The small blue circles represent the individual sensitivity and specificity coordinate points obtained at different cut-off values of the respective scoring systems on the ROC curve. The solid blue line connecting these points represents the ROC curve, illustrating the diagnostic performance of the score across varying thresholds. The diagonal straight line represents the line of no discrimination (reference line), indicating the performance expected by chance alone (area under the curve = 0.5) ASRM, American Society for Reproductive Medicine; ROC, receiver operating characteristic

As shown in Table 5, the sensitivity of the EFI score for predicting ongoing pregnancy was higher than that of the ASRM score. However, the AUC values obtained from ROC analysis for EFI and ASRM scores were comparable (0.86 and 0.87, respectively), suggesting similar predictive performance for ongoing pregnancy in the study population. The selected cut-offs for both EFI and ASRM scores in predicting clinical and ongoing pregnancy outcomes were statistically significant. For ongoing pregnancy, the p values for EFI and ASRM score cut-offs were 0.006 and <0.001, respectively. The ROC curves of EFI and ASRM scores plotted against ongoing pregnancy rates are shown in Figure 3 and Figure 4, respectively.

**Figure 5:**
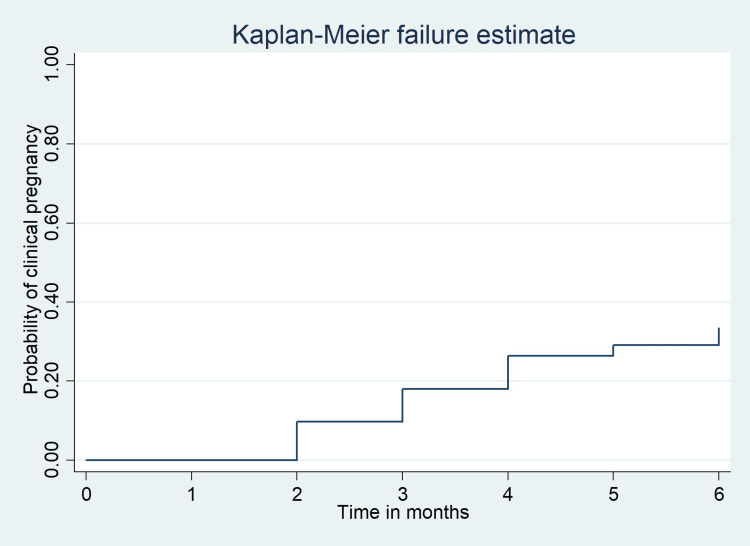
Probability of clinical pregnancy within six months after surgery for endometriosis

The probability of conception following surgery was evaluated using Kaplan-Meier analysis and plotted against time to conception, as shown in Figure 5.

The cumulative probability of conception increased progressively from approximately 0.05 to 0.30 between the second and fifth postoperative months, after which the curve demonstrated a plateau during the sixth month. Since the follow-up period in the present study was limited to six months after surgery, the probability of conception beyond this duration could not be assessed.

**Figure 6:**
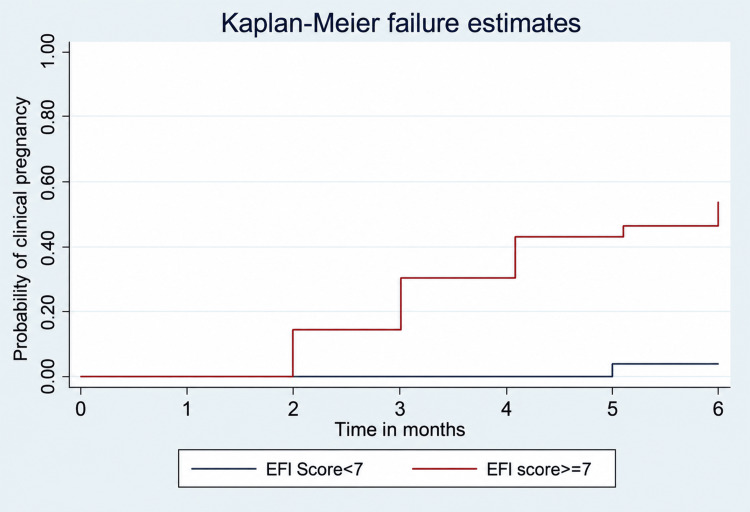
Correlation between EFI and the probability of non-ART pregnancy using EFI cut-off EFI, Endometriosis Fertility Index; non-ART, non-assisted reproductive technology

Kaplan-Meier analysis stratified according to EFI score categories (<7 and ≥7) demonstrated a significantly higher probability of conception and shorter time to pregnancy in women with EFI scores of ≥7 compared to those with EFI scores of <7, as shown in Figure 6.

These findings suggest that higher EFI scores are associated with improved non-ART pregnancy outcomes following corrective surgery for endometriosis and further support the utility of EFI as a valuable prognostic tool in women with endometriosis-associated infertility.

**Figure 7:**
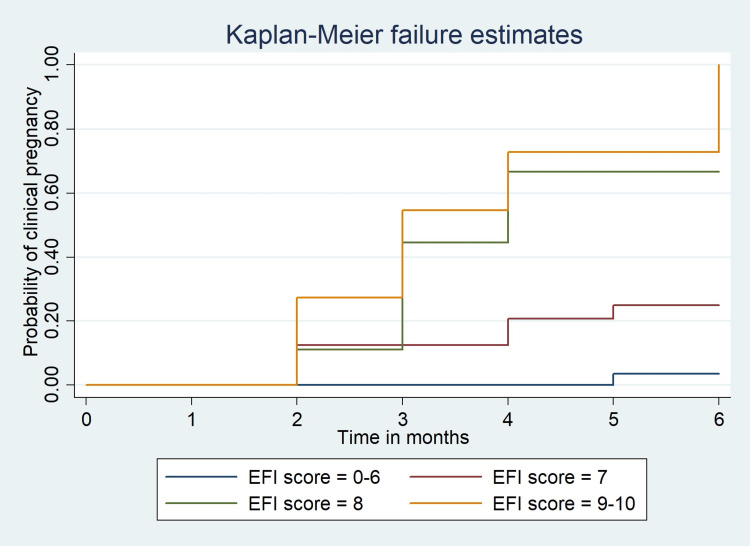
Probability of clinical pregnancy classified by EFI scores during a six-month follow-up of 72 infertile patients after surgery for endometriosis (p < 0.001) EFI: Endometriosis Fertility Index

Kaplan-Meier analysis across different EFI categories (0-6, 7, 8, and 9-10) demonstrated progressively increasing probabilities of clinical pregnancy with increasing EFI scores. Women with EFI scores of 9-10 showed the highest probability of conception, with pregnancy rates increasing between the second and sixth postoperative months and reaching maximum probability between the second and third months. In women with an EFI score of 8, the probability of clinical pregnancy increased from 0.1 at the end of the third month to 0.7 at the end of the sixth month, with the greatest increase observed during the fourth postoperative month. For patients with an EFI score of 7, the probability increased from 0.1 during the third and fourth months to 0.25 by the sixth month. Among patients with EFI scores between 0 and 6, only one achieved a clinical pregnancy during the sixth postoperative month. The comparison of Kaplan-Meier curves using the log-rank test demonstrated a statistically significant difference among EFI categories (p < 0.001), indicating that higher EFI scores were associated with better clinical pregnancy outcomes. The Kaplan-Meier curves for the different EFI categories are shown in Figure 7.

The probability of clinical pregnancy at six months following surgery for endometriosis, derived from Kaplan-Meier analysis across different EFI categories, is presented in tabulated form in Table 6.

**Table 6:** Probability of clinical pregnancy at six months in different EFI groups CI, confidence interval; EFI, Endometriosis Fertility Index

EFI score	Probability of clinical pregnancy
0-6	0.04 (95% CI: 0.23-0.01)
7	0.25 (95% CI: 0.47-0.12)
8	0.67 (95% CI: 0.92-0.38)
9-10	1.00

After establishing the optimal cut-offs for EFI and ASRM scores for predicting pregnancy outcomes in the study population, odds ratios with 95% confidence intervals were calculated for clinical pregnancy. Women with an EFI score of ≥7 had an odds ratio of 29.6 for achieving a clinical pregnancy, whereas women with an ASRM score of <21 had an odds ratio of 19.0. Both associations were statistically significant, indicating a strong relationship between these score cut-offs and the likelihood of clinical pregnancy. The odds ratios for clinical pregnancy according to EFI and ASRM score cut-offs are presented in Table 7.

**Table 7:** Odds ratio of clinical pregnancy according to cut-offs for EFI and ASRM scores Data presented as n (%). P < 0.05 is considered statistically significant EFI, Endometriosis Fertility Index; ASRM, American Society for Reproductive Medicine; CI, confidence interval

Variable	Clinical pregnancy (n = 24)	No clinical pregnancy (n = 48)	Odds ratio (95% CI)	P value
EFI score				
≥7	23 (95.8%)	21 (43.7%)	29.6 (3.7-237.1)	0.001
<7	1 (4.2%)	27 (56.2%)	Reference	-
ASRM score				
<21	20 (83.3%)	10 (20.8%)	19.0 (5.3-68.3)	<0.001
≥21	4 (16.7%)	38 (79.2%)	Reference	-

Similarly, odds ratios for ongoing pregnancy were calculated using the established EFI and ASRM score cut-offs. Women with an EFI score of ≥7 had an odds ratio of 18.7 for achieving ongoing pregnancy, which was statistically significant (p = 0.006). Likewise, women with an ASRM score of <21 had an odds ratio of 14.8, which also demonstrated statistical significance (p < 0.001). The odds ratios for ongoing pregnancy according to EFI and ASRM score cut-offs are presented in Table 8.

**Table 8:** Odds ratio of ongoing pregnancy according to cut-offs for EFI and ASRM scores Data presented as n (%). P < 0.05 is considered statistically significant EFI, Endometriosis Fertility Index; ASRM, American Society for Reproductive Medicine; CI, confidence interval

Variable	Ongoing pregnancy (n = 19)	No ongoing pregnancy (n = 53)	Odds ratio (95% CI)	P value
EFI score				
≥7	18 (94.7%)	26 (49.1%)	18.7 (2.3-150.3)	0.006
<7	1 (5.3%)	27 (50.9%)	Reference	-
ASRM score				
<21	16 (84.2%)	14 (26.4%)	14.8 (3.7-58.8)	<0.001
≥21	3 (15.8%)	39 (73.6%)	Reference	-

Cox proportional hazards regression analysis was performed to evaluate the association between different EFI score groups and time to conception following surgery for endometriosis. Women with an EFI score of ≥7 had a significantly higher likelihood of conceiving within six months after surgery compared to those with EFI scores of <7, with a hazard ratio of 19.2 (95% CI: 2.6-142.1; p = 0.004). The hazard ratio progressively increased with increasing EFI scores, indicating that higher EFI scores were associated with a shorter time to conception. Statistically significant associations were observed for EFI scores above 7. The hazard ratios for different EFI score categories are presented in Table 9.

**Table 9:** Proportional hazard analysis of EFI scores against time taken for pregnancy after surgery P < 0.05 is considered statistically significant EFI, Endometriosis Fertility Index; CI, confidence interval

EFI score	Hazard ratio (95% CI)	P value
Binary grouping		
<7	Reference	-
≥7	19.2 (2.6-142.1)	0.004
Categorical grouping		
0-6	Reference	-
7	8.1 (0.97-67.4)	0.053
8	29.4 (3.4-251)	0.002
9-10	50.5 (6.1-418.3)	<0.001

Among the 72 women included in the study, the majority had Endometriosis Fertility Index (EFI) scores ranging between 5 and 8, with 24 women (33.3%) having an EFI score of 7. Patients were categorized into five groups based on their EFI scores. Among women with EFI scores of 9-10, all 11 women (100%) achieved clinical pregnancy within six months following surgery; however, three (27.3%) experienced spontaneous abortions before 20 weeks, resulting in eight ongoing pregnancies (72.7%). Among the 33 women with EFI scores of 7-8, 12 women (36.4%) achieved clinical pregnancy, of whom two (16.7%) experienced spontaneous abortions, resulting in 10 ongoing pregnancies (30.3%). In the EFI score group of 5-6, only one patient (5.9%) achieved pregnancy within six months, whereas no pregnancies were observed among patients with EFI scores of ≤4. No ectopic pregnancies occurred in the study population. The cumulative clinical and ongoing pregnancy rates across different EFI groups within six months after surgery are presented in Table 10.

**Table 10:** EFI scores and cumulative pregnancy rate EFI: Endometriosis Fertility Index

EFI score	Number of patients	Number of patients with clinical pregnancy within six months after surgery	Number of patients with ongoing pregnancy	Number of miscarriages
0-2	1	0	0	0
3-4	5	0	0	0
5-6	22	1 (4.5)	1 (4.5)	0
7-8	33	12 (36.4)	10 (30.3)	2 (16.7)
9-10	11	11 (100)	8 (72.7)	3 (27.3)

## Discussion

This cohort study supports EFI as a strong predictor of pregnancy outcomes without assisted reproductive technologies after laparoscopic surgery in infertile women with endometriosis. Women with higher EFI scores had a higher chance of conceiving within six months, highlighting its prognostic value. The predictive accuracy of EFI in the present study was supported by high sensitivity (95.8%), area under the curve (AUC) of 0.90, and a strong hazard ratio of 19.2 for conception among women with an EFI of ≥7. Therefore, this index is a promising tool for assessing the probability of spontaneous conception after the surgical treatment of endometriosis.

This study shows that EFI offers valuable prognostic information for counselling patients on reproductive potential after surgery. Higher EFI scores link to higher pregnancy chances and faster conception, highlighting that reproductive organ health impacts outcomes in women with endometriosis.

The ASRM classification system is currently the gold standard for staging endometriosis, but studies have shown that the staging of endometriosis according to ASRM has poor prognostic value for fertility outcomes [13,17,28,29]. Because it is an anatomical classification, it does not predict postoperative reproductive function or include key fertility parameters such as infertility duration or prior pregnancy history [15,16]. Hence, ASRM alone cannot provide adequate prognostic information for fertility counselling and decision-making.

EFI incorporates reproductive parameters, filling a gap in endometriosis classification that emphasizes anatomy but neglects reproductive potential. It links postoperative adnexal function to patient characteristics, helping estimate spontaneous pregnancy and guide treatment.

The present data are consistent with the initial study by Adamson and Pasta showing increasing cumulative pregnancy rates with increasing EFI scores [13]. In that original report, the EFI became the first validated classification system for endometriosis, enabling the prediction of fertility after surgery.

Wang et al. evaluated the predictive ability of EFI in women undergoing IVF treatment following the surgical management of endometriosis and demonstrated that EFI showed better predictive performance than the ASRM classification [3]. This suggests that EFI might be applicable for predicting reproductive outcomes across various cycles. Li et al. showed that EFI may help clinicians decide the timing of assisted reproductive techniques post surgery [17]. Women with low EFI should pursue early ART, while those with higher EFI might try spontaneous pregnancy first, considering ART.

Garavaglia et al. demonstrated the predictive value of EFI in both spontaneous conception and assisted reproductive outcomes, further supporting its clinical utility [19]. Maheux-Lacroix et al. demonstrated a strong correlation between the EFI score and live birth rates following surgical treatment for moderate and severe endometriosis [30]. Their findings highlight the importance of EFI as a clinically relevant prognostic tool that can predict meaningful reproductive outcomes beyond initial conception.

A systematic review and meta-analysis published in BJOG in 2020 evaluated 17 studies including 4598 women and confirmed that EFI demonstrates moderate-to-good predictive performance for spontaneous pregnancy following endometriosis surgery [26]. The review concluded that EFI remains the most validated clinical tool for predicting non-assisted reproductive technique pregnancy outcomes and emphasized the importance of the Least Function Score as a major determinant of reproductive prognosis.

The Least Function Score represents postoperative functional status of the fallopian tubes, fimbria, and ovaries and is considered one of the most important components of EFI [13]. Previous studies have consistently demonstrated that the Least Function Score contributes significantly to the predictive ability of EFI [17,21]. The functional integrity of adnexal structures influences the chance of spontaneous conception and may explain why anatomical staging alone has limited predictive ability.

EFI offers an objective way for clinicians to estimate spontaneous conception chances after laparoscopic endometriosis surgery. Patients with higher EFI scores might benefit from expectant management or ovulation induction before assisted reproductive techniques. Conversely, patients with low EFI scores may be counselled for early referral to assisted reproductive technologies, thereby reducing delay in achieving pregnancy.

EFI may therefore assist clinicians in individualized fertility counselling and improve shared decision-making regarding treatment strategies. The use of EFI in routine clinical practice may help optimize fertility outcomes and reduce unnecessary delay in the initiation of assisted reproductive techniques.

Limitations

This study has several limitations. First, it was conducted at a single center with a relatively small sample size and only 24 clinical pregnancy events, which may have limited the precision and stability of effect estimates, including ROC-derived performance measures and hazard ratios. Second, the follow-up period was limited to six months, whereas most EFI validation studies have reported cumulative pregnancy outcomes over 12-36 months; therefore, pregnancies occurring beyond six months and live birth outcomes were not captured. Third, postoperative fertility management was individualized and included ovulation induction and/or IUI in selected patients. Details of postoperative fertility management, including expectant management, ovulation induction, and intrauterine insemination, were not systematically recorded. Therefore, the potential influence of these interventions on pregnancy outcomes and time to conception could not be assessed. Consequently, the study reflects pregnancy outcomes following surgery within a non-IVF treatment pathway rather than purely spontaneous conception, introducing the possibility of treatment-effect bias and residual confounding. Fourth, multivariable Cox regression adjusting for potential confounders was not performed because of the modest sample size and limited number of events. Fifth, the EFI cut-off of ≥7 was derived from ROC analysis within the study cohort and was not prospectively pre-specified, raising the possibility of optimism bias. Finally, the observed 100% clinical pregnancy rate in the EFI 9-10 subgroup should be interpreted cautiously because it was based on only 11 participants. Larger multicentric studies with longer follow-up, live birth assessment, standardized postoperative management, and external validation are needed to further establish the predictive performance and generalizability of EFI.

## Conclusions

The present study successfully validated the Endometriosis Fertility Index (EFI) as a predictor of non-ART pregnancy outcomes in women with endometriosis-associated infertility following laparoscopic surgery. Higher clinical and ongoing pregnancy rates were observed among women with higher EFI scores. Furthermore, life table analysis demonstrated a significant relationship between EFI score and time to conception, indicating that increasing EFI scores were associated with improved fertility outcomes following the surgical management of endometriosis.

Both EFI and r-ASRM scores demonstrated good predictive performance for clinical and ongoing pregnancy outcomes. While EFI showed higher sensitivity for identifying women likely to conceive, r-ASRM demonstrated higher specificity, indicating a trade-off between the two scoring systems. The overall discriminative ability of EFI and r-ASRM was comparable, as reflected by similar areas under the ROC curve. However, EFI offers additional clinical value by incorporating both historical and postoperative functional factors and by providing prognostic information regarding the likelihood and timing of conception following surgery. These findings support the use of EFI as a valuable fertility prognostic tool that may assist in individualized patient counselling, fertility prognostication, and decision-making regarding postsurgical fertility management strategies in women with endometriosis-associated infertility.
